# Consensus-Based Core Set of Outcome Measures for Clinical Motor Rehabilitation After Stroke—A Delphi Study

**DOI:** 10.3389/fneur.2020.00875

**Published:** 2020-09-02

**Authors:** Johannes Pohl, Jeremia Philipp Oskar Held, Geert Verheyden, Margit Alt Murphy, Stefan Engelter, Agnes Flöel, Thierry Keller, Gert Kwakkel, Tobias Nef, Nick Ward, Andreas Rüdiger Luft, Janne Marieke Veerbeek

**Affiliations:** ^1^Department of Neurology, University of Zurich and University Hospital Zurich, Zurich, Switzerland; ^2^Department of Rehabilitation Sciences, KU Leuven—University of Leuven, Leuven, Belgium; ^3^Institute of Neuroscience and Physiology, Clinical Neuroscience, University of Gothenburg, Gothenburg, Sweden; ^4^Department of Neurology and Department of Clinical Research, University of Basel, Basel, Switzerland; ^5^Neurorehabilitation Unit and University Center for Medicine of Aging and Rehabilitation, Felix Platter Hospital, University of Basel, Basel, Switzerland; ^6^Department of Neurology, University of Greifswald, Greifswald, Germany; ^7^German Center for Neurodegenerative Diseases, Greifswald, Germany; ^8^TECNALIA, Basque Research and Technology Alliance (BRTA), Neurorehabilitation Area at the Health Division, Donostia-San Sebastian, Spain; ^9^Department of Rehabilitation Medicine, Amsterdam Neuroscience and Amsterdam Movement Sciences, Amsterdam University Medical Centre, Amsterdam, Netherlands; ^10^Department Non-acquired-brain Injuries, Amsterdam Rehabilitation Centre Reade, Amsterdam, Netherlands; ^11^Gerontechnology and Rehabilitation Group, University of Bern, Bern, Switzerland; ^12^Artorg, Center for Biomedical Engineering Research, University of Bern, Bern, Switzerland; ^13^Department of Movement and Clinical Neuroscience, UCL Queen Square Institute of Neurology, London, United Kingdom; ^14^The National Hospital for Neurology and Neurosurgery, Queen Square, London, United Kingdom; ^15^cereneo, Center for Neurology and Rehabilitation, Vitznau, Switzerland

**Keywords:** stroke, motor rehabilitation, clinical, outcome measures, Delphi study

## Abstract

**Introduction:** Outcome measures are key to tailor rehabilitation goals to the stroke patient's individual needs and to monitor poststroke recovery. The large number of available outcome measures leads to high variability in clinical use. Currently, an internationally agreed core set of motor outcome measures for clinical application is lacking. Therefore, the goal was to develop such a set to serve as a quality standard in clinical motor rehabilitation poststroke.

**Methods:** Outcome measures for the upper and lower extremities, and activities of daily living (ADL)/stroke-specific outcomes were identified and presented to stroke rehabilitation experts in an electronic Delphi study. In round 1, clinical feasibility and relevance of the outcome measures were rated on a 7-point Likert scale. In round 2, those rated at least as “relevant” and “feasible” were ranked within the body functions, activities, and participation domains of the *International Classification of Functioning, Disability, and Health* (*ICF*). Furthermore, measurement time points poststroke were indicated. In round 3, answers were reviewed in reference to overall results to reach final consensus.

**Results:** In total, 119 outcome measures were presented to 33 experts from 18 countries. The recommended core set includes the Fugl–Meyer Motor Assessment and Action Research Arm Test for the upper extremity section; the Fugl–Meyer Motor Assessment, 10-m Walk Test, Timed-Up-and-Go, and Berg Balance Scale for the lower extremity section; and the National Institutes of Health Stroke Scale, and Barthel Index or Functional Independence Measure for the ADL/stroke-specific section. The Stroke Impact Scale was recommended spanning all *ICF* domains. Recommended measurement time points are days 2 ± 1 and 7; weeks 2, 4, and 12; 6 months poststroke and every following 6th month.

**Discussion and Conclusion:** Agreement was found upon a set of nine outcome measures for application in clinical motor rehabilitation poststroke, with seven measurement time points following the stages of poststroke recovery. This core set was specifically developed for clinical practice and distinguishes itself from initiatives for stroke rehabilitation research. The next challenge is to implement this clinical core set across the full stroke care continuum with the aim to improve the transparency, comparability, and quality of stroke rehabilitation at a regional, national, and international level.

## Introduction

Despite the advances of primary and secondary prevention and the availability of acute medical interventions, stroke remains the second most common cause of disability worldwide (1). Because of an aging population and increasing rates of stroke in younger adults, the number of stroke cases is most likely to increase to 1.5 million cases by the year 2025 (2). In respect to resulting challenges to national health systems and social economy, a European Stroke Action Plan was formulated and includes the domains *primary prevention, organization of stroke services, management of acute stroke, secondary prevention, rehabilitation, evaluation of stroke outcome/quality assessment*, and *life after stroke* (3). As motor deficits due to stroke lead to limitations in the performance of activities of daily living (ADL), reduced societal participation, and a lower quality of life (4), outcome measures (OMs) in the motor domain comprise a key role in optimizing and monitoring attainable treatment goals and providing transparency regarding the quality along the stroke care continuum (5). An early and systematic administration of OMs could have multiple benefits for clinicians and patients, such as objective monitoring of the recovery process and the facilitation of goal-oriented interprofessional collaboration, and to support the stroke survivor's education. Currently, a significant number of OMs are available for different clinical settings and stages poststroke (6). Consequently, there is a large variability in clinical use, which hampers transparency and the comparability of motor rehabilitation within and across countries.

Clinical guidelines for evidence-based practice regarding stroke operate on a national level and lack international consensus regarding the use of OMs and, more importantly, the timing of measurements. Despite attempts of implementing the evidence resulting from stroke rehabilitation research into clinical stroke rehabilitation by specific clinical guidelines, the adherence across Europe is often insufficient (7). Standards for OMs to use are not commonly practiced, and the administration of OMs in the field of stroke rehabilitation and other areas is surprisingly low (8). Recently, an international group of researchers systematically reviewed existing clinical guidelines on recommendations for upper extremity assessments and concluded that there is a lack of explicit recommendations on OMs in most of the guidelines (9).

Specifically for research purposes, consensus-based recommendations for sensorimotor measurements in stroke rehabilitation trials were developed by the international Stroke Recovery and Rehabilitation Roundtable (SRRR) to set standards for methodological quality for clinical trials on the body functions and activities domains of the *International Classification of Functioning, Disability, and Health* (*ICF*) (10). Also, local and national research groups recommended OMs for stroke research (11–13), including specific interventions such as robotics (14), a single poststroke recovery stage (15), and patient-reported outcomes only (16, 17). Although these efforts are very valuable for stroke rehabilitation research, the recommendations cannot be translated one to one into clinical practice, as the requirements on OMs for clinical use might differ by aspects of the administration time, the number of measurement time points, and the length of follow-up. It is also likely that, for clinical practice, a broader spectrum in terms of impairment and disability levels as well as body sections is relevant, when compared to those covered by various research initiatives. Furthermore, the clinical core set should incorporate the patient's multidomain perspective (18) that was not covered by the SRRR research recommendations, and an international group of clinical stakeholders should be involved.

Therefore, the aim of the present study was to develop an international consensus-based core set of OMs with fixed measurement time points for clinical use in motor rehabilitation after stroke, which is relevant for the full stroke rehabilitation pathway. This set is a key ingredient for transparent stroke rehabilitation and allows alignment between regions and countries with the ultimate goal to improve stroke patients' motor outcomes.

## Materials and Methods

### Identification of Outcome Measures

An initial collection of sensorimotor OMs was compiled based on an extensive search in relevant systematic reviews (6, 10–27), clinical guidelines (28, 29), and electronic rehabilitation measurement databases [e.g., StrokEngine (30) and Shirley Ryan Ability Lab (31)] by two researchers (JP, JV). The OMs had to meet the following inclusion criteria: (1) assess the motor domain, (2) validated for use in stroke patients, and (3) have a good reliability for the stroke population (intraclass correlation coefficient > 0.7). Eligible OMs were allocated to one of the following three sections: the upper extremity, lower extremity, or ADL/stroke-specific section. The constructs of trunk control, balance, and mobility were assigned to the lower extremity section. The ADL/stroke-specific section included a broader variety of constructs, assessing stroke-specific motor-related functions, activities, and participation. Within each section, OMs were classified according to the *ICF* domains body structure and function, activities, and participation (18).

### Delphi Study Design

A Delphi study design was used to develop the consensus-based core set. The three-round Delphi study was conducted from November 2018 until April 2019. Per section, we aimed to have one OM in each *ICF* domain that could be applied, regardless of stroke severity. In the lower extremity section, one OM per *ICF* domain had to be applicable for both patients with and without walking ability.

**Figure 1:**
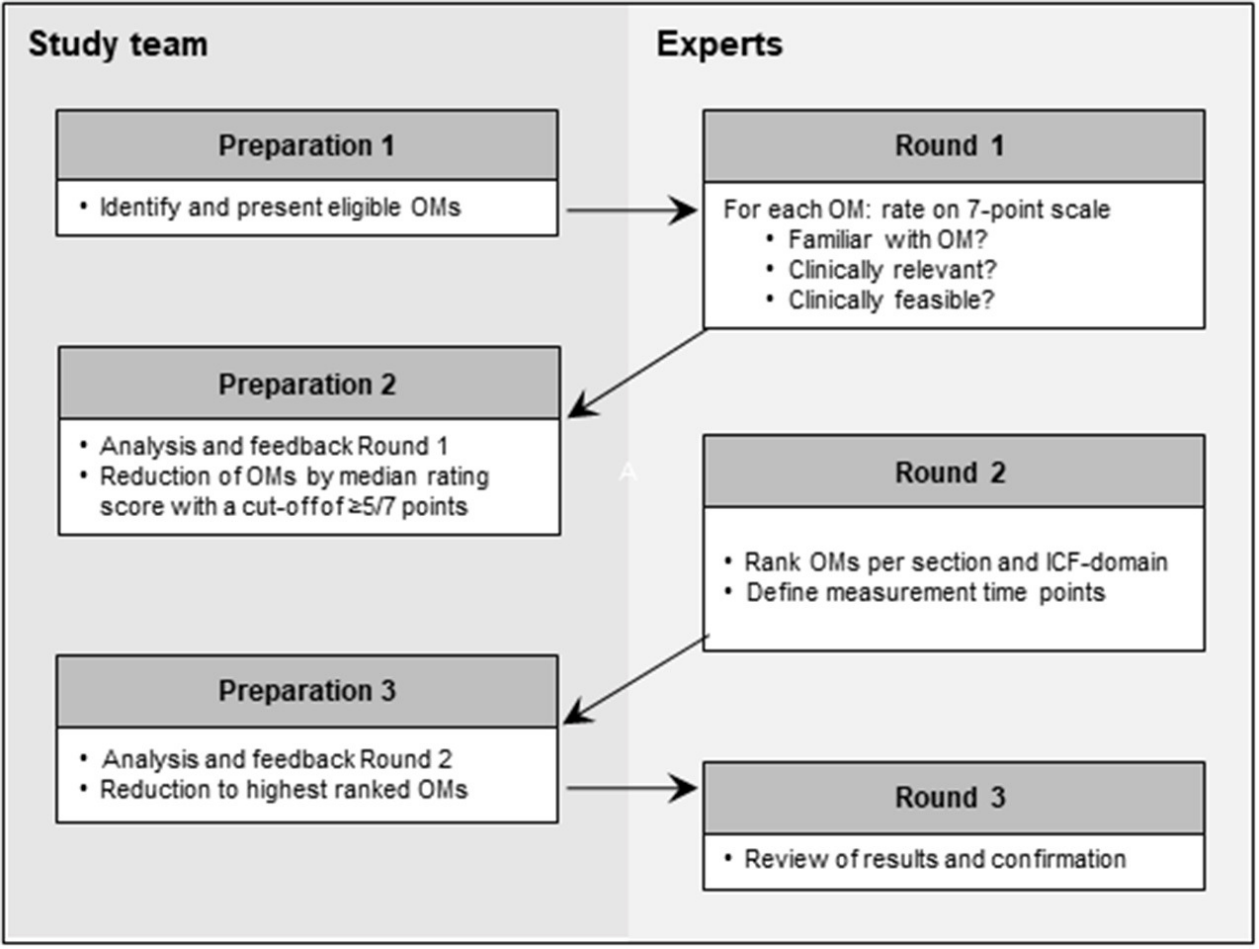
Overview of the Delphi process. *ICF, International Classification of Functioning, Disability, and Health*; OMs, outcome measures.

After each round, each expert received an individualized feedback report with details of the previous round's results in reference to their personal rating. In round 1, each OM was presented with details of the measure's construct, costs, time to administer, and clinimetric properties [validity, reliability, and minimal clinically important difference (MCID)] in line with COSMIN recommendations (Figure 1) (32). For each OM, experts had to rate on a 7-point Likert scale: (1) how familiar they were with that measure, (2) its relevance for clinical practice, and (3) its clinical feasibility. The initial set of OMs was then reduced to those, rated with scores of at least five of seven points for both clinical relevance and clinical feasibility. In round 2, the reduced set of OMs had to be prioritized for each section and *ICF* domain by assigning ranks in ascending order. As for some lower extremity OMs, patients need to be able to walk; a second measure was allowed if the OM ranked first requires walking ability. For each section, the highest-ranked OM within each *ICF* domain was included in a preliminary core set for the third round. Additionally, the experts designed a specific measurement scheme, indicating their preferred measurement time points poststroke: Within the acute phase (days 1, 3, and 7), early subacute phase (weeks 2, 4, 6, and 10), the late subacute phase (weeks 12, 16, and 20 and month 6) and for the chronic phase (every 3rd and 6th month following). The minimal agreement rate on measurement timing in round 2 was set to at least 50% ± 2%. In round 3, the experts reviewed the aggregated results presented next to their individual rankings and suggested time points and confirmed their agreement. The cutoff rate for minimal agreement on measurement time points in round 3 was 70 ± 5%.

A clearance certificate for this study was provided by the cantonal ethics committee Zurich (BASEC Nr. Req-2018-00601). Informed consent of the participating experts was not needed.

### Rehabilitation Experts

From September to October 2018, personal enquiries were sent to renowned experts of stroke rehabilitation research and with a networking approach via the following organizations: European Stroke Organization, Council of Occupational Therapists in European Countries, Research in Occupational Therapy and Occupational Science, European Network Occupational Therapy, and the European Network of Physiotherapy in Higher Education. It was our goal to recruit a balanced group of international experts with different clinical backgrounds, including medical doctors, physical therapists, occupational therapists, and rehabilitation engineers. Persons were considered eligible if they had expertise in clinical stroke rehabilitation and clinical research or rehabilitation engineering research and hold at least a master of science degree. The experts were kept ignorant about the other participating experts and received no compensation.

### Data Collection and Analysis

The participants received detailed information and instructions on a website with access to the first round's electronic survey created with Research Electronic Data Capture (REDCap, Vanderbilt University Medical Center, USA). Rounds 2 and 3 were carried out via personalized electronic forms, sent and responded via email. Responses were filed by hand (JP) and cross-checked for insertion errors (JH, JV). Feedback of results was given after each round, and equivocal responses were followed up by inquiries via email. Rankings and ratings were analyzed as medians and interquartile ranges. The data were analyzed after each round and presented for the next round. Data analysis and visualization were conducted with Microsoft Office Professional Plus 2016 (Microsoft Cooperation, Redmond, WA, USA).

## Results

### Participants

Written inquiries yielded 46 eligible experts of whom 33 experts from 18 countries participated and completed the first round of the Delphi study with a response rate of 72% (Table 1). Final agreement was given by 27 experts with three participants lost after the first round and three after the second round.

**Table 1:** Participant characteristics.

**Characteristic**	***N* = 33**
**Profession**, ***n*** **(%)**	
Medical doctor	12 (36.4)
Occupational therapist	8 (24.2)
Physical therapist	11 (33.3)
Rehabilitation engineer	2 (6.1)
**Experience, Median (IQR), Years**	
Clinical	15 (9)
Research	15 (14)
**Region of practice**	Austria, Belgium, Czech Republic, Cyprus, Denmark, Finland, Germany, Italy, Latvia, Lithuania, the Netherlands, Norway, Poland, Portugal, Spain, Sweden, Switzerland, United Kingdom

*IQR, interquartile range*.

### Development of the Core Set for Clinical Motor Rehabilitation After Stroke

**Figure 2:**
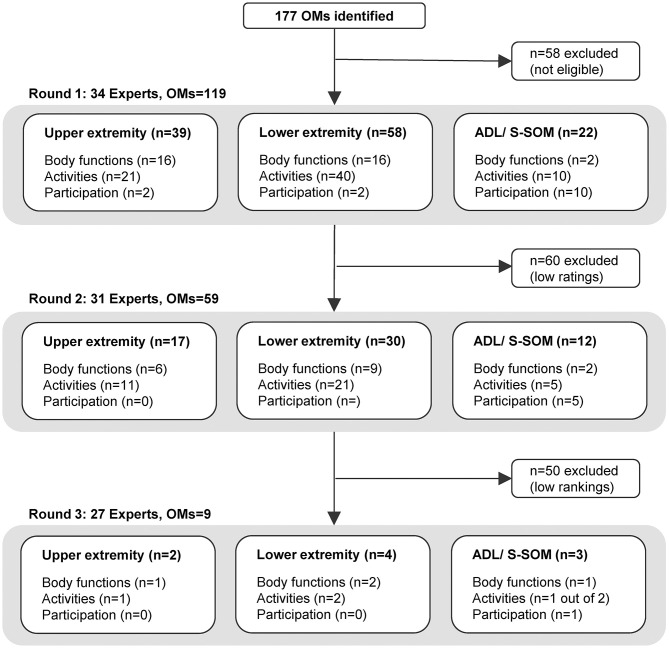
Flowchart of outcome measures per Delphi round, section, and *ICF* domain. ADL, activities of daily living; *ICF, International Classification of Functioning, Disability, and Health*; OMs, outcome measures.

In total, 177 OMs were identified, of which 119 met the inclusion criteria and were presented to the experts. Fifty-nine OMs were rated as being relevant and feasible and were consecutively ranked in round 2. In round 3, final agreement for a core set of nine OMs was given (Figure 2, Table 2).

**Table 2:** Core set of outcome measures for clinical motor rehabilitation after stroke.

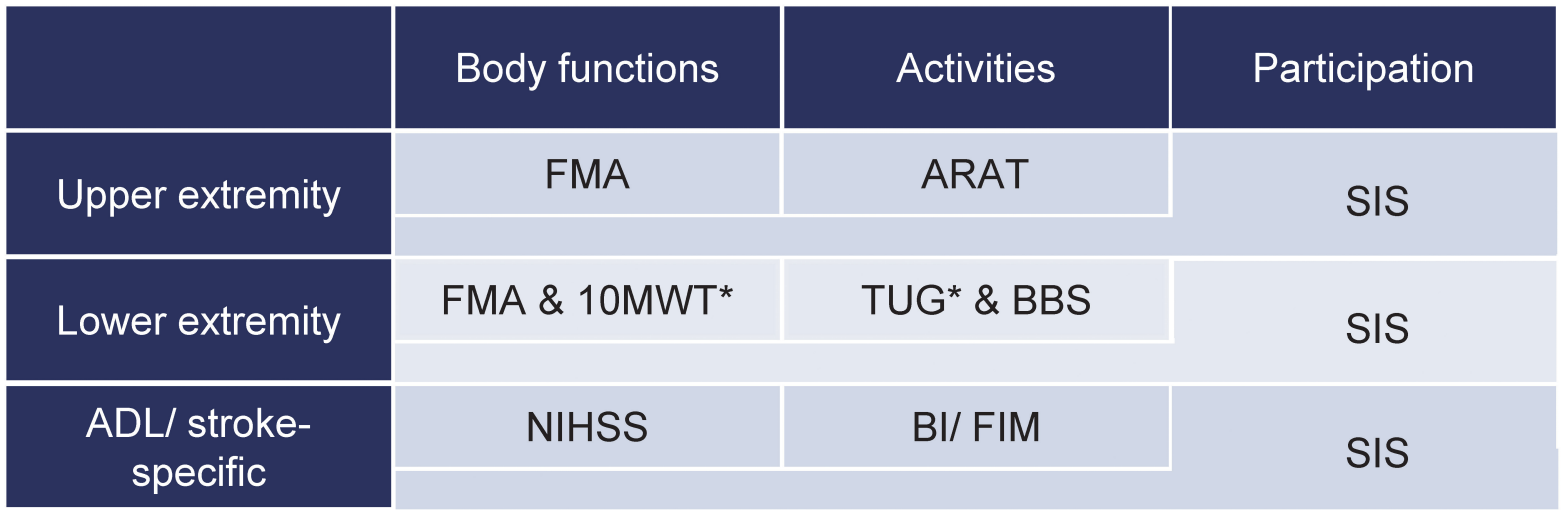

*Measure only required for patients with a Functional Ambulation Categories score of ≥3/5.

*ADL, activities of daily living; ICF, International Classification of Functioning, Disability, and Health*.

The highest ranking in the upper extremity section was given to the Upper Extremity Subscale of the Fugl–Meyer Motor Assessment (FMA-UE) in the body functions domain and the Action Research Arm Test (ARAT) for the activities domain. In the lower extremity section, the two OMs with the highest rankings in the body functions domain were the Fugl–Meyer Motor Assessment Lower Extremity Subscale (FMA-LE) and the 10-m Walk Test (10 MWT); for the activities domain, the Timed-Up-and-Go (TUG) and the Berg Balance Scale (BBS) were indicated. Regarding the ADL/stroke-specific section, the highest ranks were given to the National Institutes of Health Stroke Scale for the body functions domain, and the Barthel Index (BI) or Functional Independent Measure (FIM) for the activities domain. Within all sections, the Stroke Impact Scale (SIS) was prioritized first for the participation domain. However, it should be noted that the SIS also provides the patient's perspective on the body functions and activities domains of the *ICF*. Subsections of the SIS (hand function, mobility, strength) were presented separately for the upper extremity and lower extremity sections and the whole SIS for the ADL/stroke-specific section. Detailed rankings of the OMs per section and *ICF* domain and details of the OMs, such as clinimetric properties with references and measurement protocols, can be found in the online supplement (Tables S1–S3, S6–S8, respectively).

### Measurement Time Points

In round 2, between three and eight measurement time points per OM within *ICF* domains were proposed by the experts showing consistent agreement in a range from 48 to 90%. Agreement rates for measurements at 6, 10, 12, and 20 weeks were below 50 ± 2% and were not presented in the last round. Because of expert comments, the first two time points were combined and presented in the final round as one measurement to be administered within the first 3 days. Final agreement was given, with agreement rates ranging from 65.2 to 91.3% for a maximum of seven measurement time points for the upper and lower extremity body functions domain to be taken between days 1 and 3; at day 7; at weeks 2, 4, and 12; at 6 months; and every following 6th month. In the activities domain of the upper and lower extremity sections, agreement of measurement time points followed the same scheme but starting at day 7. A deviating scheme was compiled for the ADL/stroke-specific OMs (Table 3). Final agreement rates can be found in the online supplement (Tables S4, S5).

**Table 3:** Measurement time points of the core set for clinical motor rehabilitation after stroke.

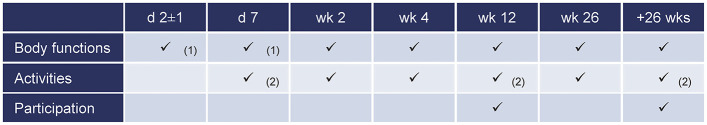

*✓, recommended time point for assessment; d, day; m, month; wk, week; (1) exceptional time points for the National Institutes of Health Stroke Scale, only indicated at these time points; (2) exceptional time points for the Barthel Index/Functional Independence Measure, only indicated at these time points*.

## Discussion

The goal of this Delphi study was to develop a core set of OMs for clinical motor rehabilitation after stroke as a tool to evaluate the quality of stroke rehabilitation at a local, national, and international level. The consensus-based core set contains nine OMs that cover a wide range of measurement constructs within all *ICF* domains that are applicable to patients with different stroke severity levels. In addition, a framework with fixed measurement time points was established, following a non-linear pattern (33), with more frequent measurements within the first 3 months after stroke and larger measurement intervals in the chronic phase. The core set was developed on the basis of independent opinions of international experts of different health care professions. All experts have comprehensive experience in clinical stroke rehabilitation. This active involvement of clinical stakeholders ensures the set's clinical relevance, feasibility, and applicability.

### Core Set for Clinical Motor Rehabilitation After Stroke

The FMA-UE and ARAT are the selected OMs for the upper extremity section and are in line with the minimal set developed for stroke rehabilitation trials (10). Both instruments have excellent clinimetric properties and therefore demonstrate a high measurement quality for clinical stroke rehabilitation (6, 11). In order to guarantee consistent measurements that allow comparing clinical and research findings, standardized measurement procedures should be followed.

The lower extremity section of the core set covers a large variety of constructs within the spectrum of body functions and activities: motor function, gait speed, functional mobility, and balance in sitting and standing. The OMs are feasible and relevant for stroke patients with and without walking ability. Outcome measures on the activities domain are discriminated by the Functional Ambulation Categories (FAC). Hence, the FAC is not *per se* included as one of the core set's OMs, but it is a screening tool to determine which OMs should at least be applied. The constructs of motor function (FMA-LE) and balance (BBS) should be evaluated in all patients, whereas walking speed (10 MWT) and functional mobility (TUG) should only be assessed in patients with an FAC score of at least three out of five. Comparing these OMs for the lower extremity with those recommended for stroke rehabilitation research (10) clearly shows that although the constructs of functional balance and mobility were not recommended for research, they are found to be relevant for the clinical setting.

The ADLs/stroke-specific OMs section covers the constructs stroke severity (body functions domain) and basic ADLs (activities domain). Within the activities domain, the highest rank is shared by the BI and FIM, which are highly correlated (*r* = 0.92–0.99) (34). These OMs can be chosen upon individual considerations within stroke services. The FIM requires annual license fees and provides chargeable access to training materials, offers data services, and contains additional socio-cognitive items. The BI might be favorable regarding time and financial resources (online supplement, Table S8).

The majority of the recommended OMs are designed to objectify the patient's observed functional impairment or to evaluate motor capacity in a standardized test environment, which is defined as the “maximum potential of an individual to succeed in the performance of a motor skill” (35). The included capacity measures are complemented by the patient-reported SIS, which is sensitive to change (36). The SIS not only covers the participation domain of the *ICF*, but also provides the patient's perspective on the body functions and activities domain. With that, it adds an important multidomain perspective to this clinical core set, a perspective on which no consensus was found for stroke rehabilitation research (10).

The core set's OMs are part of the few clinical guidelines that gave specific recommendations on OMs (9), which potentially facilitates implementation at a national level. The responsibility of clinical assessments should be shared by the involved health care professions according to their specialization. The total time to complete the core set lies between 60 and 75 min, depending on the patient's ability to understand and answer questions or to perform the required tasks of the BI or FIM.

### Measurement Time Points

The core set provides a refined framework of fixed measurement time points poststroke, with more frequent measurements early after symptom onset and a low frequent monitoring pattern in the chronic phase. This is in accordance with the logarithmic pattern of sensorimotor recovery after stroke, in which the greatest changes on the body functions and activities domains occur within the first 12 weeks after symptom onset (37, 38). In this period, behavioral restitution takes place, and thereafter, changes occur predominantly due to compensational mechanisms, reaching a plateau between ~3 and 6 months poststroke (33). Low-frequent assessment in the chronic phase allows for monitoring the patient's impairments and disabilities. In case of presence or lack of clinically relevant changes, rehabilitation can be restarted, continued, adapted, or completed (29).

The core set's seven consensus-based measurement time points are in line with existing recommendations in national clinical guidelines (29, 39) and stroke rehabilitation research guidelines (10) to assess in all four recovery phases poststroke (40). However, the experts recommended more measurement time points in the subacute phase, when compared to research recommendations. This will provide more detail about the individual motor recovery pattern across different *ICF* domains and therefore promote personalized rehabilitation and support appropriate discharge and adaptive planning regarding the home environment. The experts did not select admission and discharge as recommended measurement time points. Although we did not investigate the reason for not selecting specific time points by the experts, we hypothesize that this could be explained by the large international variability in both the length of stay and the accessibility to acute clinics and rehabilitation facilities (41). These arbitrary time points impede the comparability on a regional, national, and international level. As the consensus-based time points are a minimum number of required measurement time points, measurements at admission and discharge could be optionally implemented in the local framework to facilitate rehabilitation goal setting and evaluation.

For clinical practice, an important clinimetric requirement of OMs is their responsiveness to clinically meaningful differences. As it is known that these differences depend on the recovery phase poststroke (42), changes between measurements should be related to the poststroke phase-specific MCID. Although results of anchor-based MCIDs were no inclusion criterion for the preselection of OMs in the Delphi study, MCIDs are available from the acute to the chronic stage for most OMs (online supplement Tables S6–S8).

The experts' consensus resulted in a clear measurement pattern for upper and lower extremity body functions and activities. They proposed a scheme with less measurement time points for the ADL/stroke specific section, possibly because these OMs are not valid and responsive at all time points.

### Limitations

There are considerations to be made regarding the developed core set for clinical motor rehabilitation after stroke. First, the availability of validated translations and transcultural validations was no inclusion criterion for OMs. However, with the call for international quality standards in stroke care (3), it should be the interest of stroke services on a regional and national level, to allow for translated and validated versions of the core set's OMs. Second, although we aimed for a well-balanced group of experts in terms of clinical background, occupational therapists were underrepresented. It is unlikely that this influenced the final core set, as there were only marginal variations in the rankings between professions. Regarding the balance by regions of practice, Eastern European countries were underrepresented. Third, there is variability in agreement rates of measurement time points after rounds 2 and 3. However, there was a clear difference in agreement rates between the excluded and final recommended measurement time points. Finally, although most of the experts are still clinically active in stroke rehabilitation, many of them are also involved in research, and they may have ranked OMs using both their clinical and research experiences.

### Future Directions

In a next step, the core set for clinical motor rehabilitation after stroke should be implemented across the whole stroke care pathway, including stroke units and acute hospitals, rehabilitation facilities, and outpatient centers or private practices. It should be acknowledged that the implementation of standardized tests in the clinic is challenging. Bland and colleagues (43) demonstrated differences in adherence between settings and professions. Especially in the outpatient facilities, standardized assessments were less frequently applied. However, implementation projects have demonstrated that educational programs and assessment training leads to a successful implementation of stroke OMs in clinical practice (44), and these should be taken as a good example. Routinely scheduled time slots for fixed measurement time points could support time and resource efficiency. A reevaluation of the core set's OMs and the adherence of health care professionals to apply this set should be initiated in 5 years. The measurements' results should be fed to national registries to gain insight into the quality of clinical motor rehabilitation in the acute, subacute, and chronic phase poststroke and provide input for actions for improvement. Last but not least, a collaboration of clinicians and researchers should aim for the development of a minimal set of OMs for other important domains in clinical stroke rehabilitation, such as cognition and speech.

## Conclusion

The consensus-based core set of OMs for clinical motor rehabilitation after stroke contains nine OMs that cover the main impairments in body functions, activities, and participation on the motor domain and is complementary to recommendations for stroke rehabilitation research. Measurements should be performed at six time points within the first 6 months poststroke, and consecutive monitoring should take place every 6th month in the chronic stage. The core set and its measurement framework should be implemented throughout the whole stroke care continuum and allows benchmarking, with the long-term goal to optimize the quality of poststroke rehabilitation.

## Data Availability Statement

The datasets generated for this study are available on request to the corresponding author.

## Ethics Statement

Ethical review and approval was not required for the study on human participants in accordance with the local legislation and institutional requirements (BASEC Nr. Req-2018-00601). Written informed consent from the participants was not required to participate in this study in accordance with the national legislation and the institutional requirements.

## Author Contributions

The idea was conceived by AL, JH, and JV. JV designed the study. JP and JV searched the literature for relevant OMs and designed the electronic survey and forms. JP analyzed the responses, drafted, and edited the manuscript that was critically reviewed by JH, GV, AL, and JV. JH and JV reviewed the data entry and analysis done by JP. MA, SE, AF, TK, GK, and TN also participated in the study. All authors approved the study plan, methods, study design, critically read, and approved the final version.

## Conflict of Interest

The authors declare that the research was conducted in the absence of any commercial or financial relationships that could be construed as a potential conflict of interest.
