# Corrigendum: Consensus-Based Core Set of Outcome Measures for Clinical Motor Rehabilitation After Stroke—A Delphi Study

**DOI:** 10.3389/fneur.2021.697935

**Published:** 2021-05-27

**Authors:** Johannes Pohl, Jeremia Philipp Oskar Held, Geert Verheyden, Margit Alt Murphy, Stefan Engelter, Agnes Flöel, Thierry Keller, Gert Kwakkel, Tobias Nef, Nick Ward, Andreas Rüdiger Luft, Janne Marieke Veerbeek

**Affiliations:** ^1^Department of Neurology, University of Zurich and University Hospital Zurich, Zurich, Switzerland; ^2^Department of Rehabilitation Sciences, KU Leuven—University of Leuven, Leuven, Belgium; ^3^Institute of Neuroscience and Physiology, Clinical Neuroscience, University of Gothenburg, Gothenburg, Sweden; ^4^Department of Neurology and Department of Clinical Research, University of Basel, Basel, Switzerland; ^5^Neurorehabilitation Unit and University Center for Medicine of Aging and Rehabilitation, Felix Platter Hospital, University of Basel, Basel, Switzerland; ^6^Department of Neurology, University of Greifswald, Greifswald, Germany; ^7^German Center for Neurodegenerative Diseases, Greifswald, Germany; ^8^TECNALIA, Basque Research and Technology Alliance (BRTA), Neurorehabilitation Area at the Health Division, Donostia-San Sebastian, Spain; ^9^Department of Rehabilitation Medicine, Amsterdam Neuroscience and Amsterdam Movement Sciences, Amsterdam University Medical Centre, Amsterdam, Netherlands; ^10^Department Non-acquired-brain Injuries, Amsterdam Rehabilitation Centre Reade, Amsterdam, Netherlands; ^11^Gerontechnology and Rehabilitation Group, University of Bern, Bern, Switzerland; ^12^Artorg, Center for Biomedical Engineering Research, University of Bern, Bern, Switzerland; ^13^Department of Movement and Clinical Neuroscience, UCL Queen Square Institute of Neurology, London, United Kingdom; ^14^The National Hospital for Neurology and Neurosurgery, Queen Square, London, United Kingdom; ^15^cereneo, Center for Neurology and Rehabilitation, Vitznau, Switzerland

**Keywords:** stroke, motor rehabilitation, clinical, outcome measures, Delphi study

In the original article, there was a mistake in Table 2 as published. Important asterisks that were explained in the tables caption were not inserted in the table (second row, second and third columns). There is also an incorrect abbreviation (FMMA instead of FMA, in the second row, second column). The corrected Table 2 appears below.

**Table 2:**
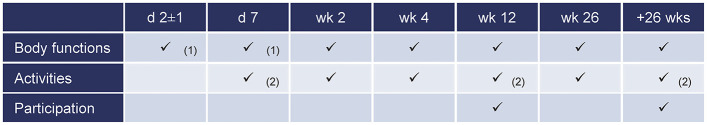
Core set of outcome measures for clinical motor rehabilitation after stroke.

*Measure only required for patients with a Function Ambulation Categories score of ≥3/5. ADL, activities of daily living; ICF, International Classification of Functioning, Disability, and Health.

In the original article, there was a mistake in Table 3 as published. Important footnotes (1) and (2), that were further explained in the table's caption, were not inserted in the table. The corrected Table 3 appears below.

**Table 3:**
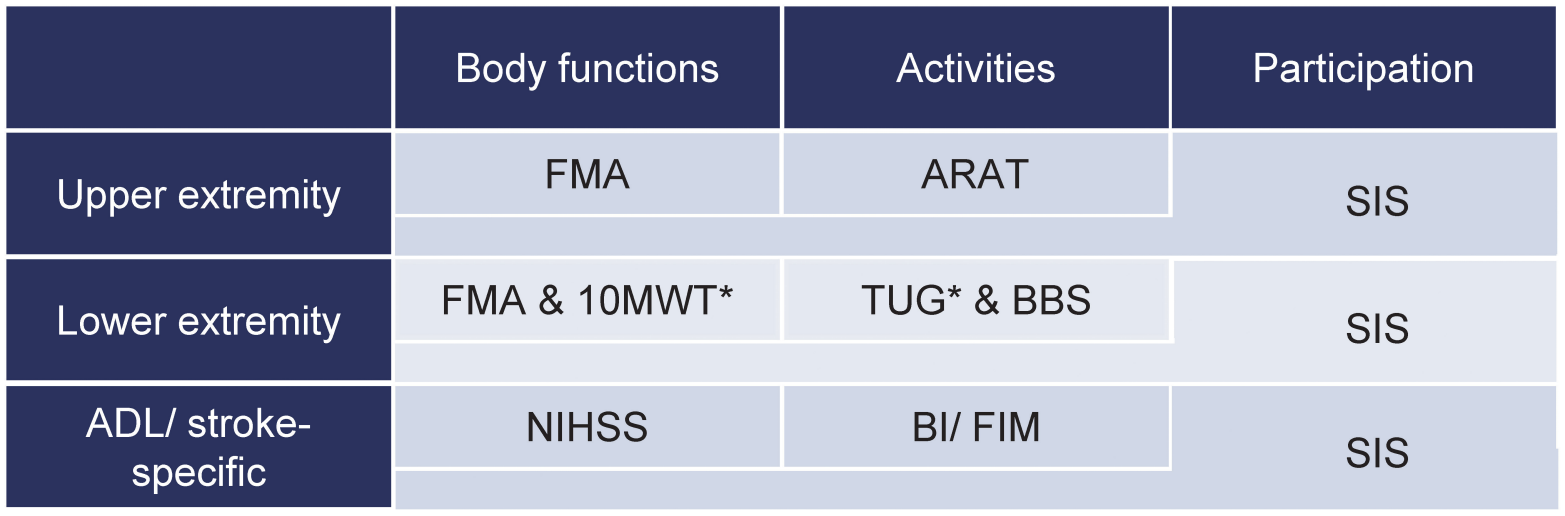
Measurement time points of the core set for clinical motor rehabilitation after stroke.

*✓, recommended time point for assessment; d, day; m, month; wk, week; (1) exceptional time points for the National Institutes of Health Stroke Scale, only indicated at these time points; (2) exceptional time points for the Barthel Index/Functional Independence Measure, only indicated at these time points*.

The authors apologize for these errors and state that they do not change the scientific conclusions of the article in any way. The original article has been updated.

