# Supplementary material for: Consensus-Based Core Set of Outcome Measures for Clinical Motor Rehabilitation After Stroke—A Delphi Study
Source: Front Neurol. 2020 Sep 2;11:875. doi: 10.3389/fneur.2020.00875 (PMC7496361; doi:10.3389/fneur.2020.00875)
Supplement: Supplementary file 1 [file Data_Sheet_1.PDF]

SUPPLEMENTARY MATERIAL

Consensus-based Core Set of Outcome Measures for Clinical Motor Rehabilitation after Stroke - A Delphi Study

Johannes Pohl[1,2], Jeremia Philipp Oskar Held[1], Geert Verheyden[2], Margit Alt Murphy[3], Stefan Engelter[4,5], Agnes Flöel[6,7], Thierry Keller[8], Gert Kwakkel[9,10], Tobias Nef[11,12], Marion Walker[13], Nick Ward[14,15], Andreas Rüdiger Luft[1,16], Janne Marieke Veerbeek[1*]

[1] University of Zurich and University Hospital Zurich, Department of Neurology, Zurich, Switzerland
[2] Department of Rehabilitation Sciences, KU Leuven – University of Leuven, Leuven, Belgium
[3] University of Gothenburg, Institute of Neuroscience and Physiology, Clinical Neuroscience, Gothenburg, Sweden
[4] University of Basel, Department of Neurology and Department of Clinical Research, Basel, Switzerland
[5] University of Basel, Felix Platter Hospital, Neurorehabilitation Unit and University Center for Medicine of Aging and Rehabilitation, Basel, Switzerland
[6] University of Greifswald, Department of Neurology, Greifswald, Germany
[7] German Center for Neurodegenerative Diseases, Greifswald, Germany
[8] TECNALIA, Basque Research and Technology Alliance (BRTA), Neurorehabilitation Area at the Health Division, San Sebastian, Spain
[9] Amsterdam University Medical Centre, Department of Rehabilitation Medicine, Amsterdam Neuroscience and Amsterdam Movement Sciences, Amsterdam, Netherlands
[10] Amsterdam Rehabilitation Centre Reade, Department Non-Acquired-Brain Injuries, Amsterdam, Netherlands
[11] University of Bern, Gerontechnology and Rehabilitation Group, Bern, Switzerland
[12] Artorg, Center for Biomedical Engineering Research, University of Bern, Bern, Switzerland
[13] University of Nottingham, Division of Rehabilitation, Ageing and Wellbeing, Nottingham, United Kingdom
[14] Department of Movement and Clinical Neuroscience, UCL Queen Square Institute of Neurology, London, United Kingdom
[15] The National Hospital for Neurology and Neurosurgery, Queen Square, London
[16] cereneo, Center for Neurology and Rehabilitation, Vitznau, Switzerland

Correspondence:

Dr. Janne Marieke Veerbeek

janne.veerbeek@usz.ch

Table of contents

1. Hierarchical ranking of outcome measures and agreed measurement time points
   - Table I: Rank list of upper extremity outcome measures
   - Table II: Rank list of lower extremity outcome measures
   - Table III: Rank list of Activities of Daily Living/ stroke-specific outcome measures

2. Agreed time points or measurements poststroke
   - Table IV: Agreement rates for measurement time points for the upper and lower extremity outcome measures by ICF-domain
   - Table V: Agreement rates for measurement time points for the Activities of Daily Living/ stroke-specific outcome measures by ICF-domain

3. Core set's details and clinimetric properties tested in stroke
   - Table VI: Details of the outcome measures for the upper extremity section
   - Table VII: Details of the outcome measures of the lower extremity section
   - Table VIII: Details of the outcome measures of the Activities of Daily Living/ stroke-specific section

4. References

1. Hierarchical ranking of outcome measures and agreed measurement time points

Table I: Rank list of upper extremity outcome measures

Outcome measure	Sum of ranks	Median rank	IQR
**Body functions**			
Fugl-Meyer Motor Assessment Upper Extremity Subscale	49.5	1	1
Fugl-Meyer Assessment Sensory Subscale	101.5	3	3
Motricity Index Upper Extremity Subscale	102	3	2.75
Grip & Pinch Dynamometry/ Handheld Dynamometry	108	3	1.75
(Modified) Ashworth Scale	129	4	1
Range of Motion	139	5	2
**Activities**			
Action Research Arm Test	82	1	3.5
Box and Block Test	122	3	3.75
Nine-Hole Peg Test	120	4	1.75
Wolf Motor Function Test	173	5.5	5
Motor Activity Log	188	6	4
Motor Assessment Scale for the Upper Extremity	200	6.5	2.75
ABILHAND	203.5	7	6
Accelerometers	206	7	4.75
Rivermead Motor Assessment Arm Section	214	8	3.5
Chedoke Arm Hand Activity Inventory	230.5	8.5	2.75
Frenchay Arm Test	232	9	3
**Participation**			
Outcome measures on the participation level were rated within the Activities of Daily Living/ stroke-specific section, see Table III			

Legend: ADL, Activities of Daily Living; IQR, Interquartile Range.

Table II: Rank list for lower extremity outcome measures

Outcome measure	Sum of ranks	Median rank	IQR
**Body functions**			
Fugl-Meyer Motor Assessment Lower Extremity Subscale	84	2	4.75
10-Meter Walk Test	81.5	2.5	2.75
Motricity Index Lower Extremity Subscale	113.5	4	3
6-Minute Walk Test	127.5	4.5	4
Fugl-Meyer Assessment Sensory Subscale	129	4.5	4.63
Rivermead Motor Assessment	130	5.5	3.88
Range of Motion	154.5	6	3
Composite Spasticity Scale	175	6.75	2.75
Myometer Muscle Strength Reading	173	7	2
**Activities (FAC 0-5)**			
Timed-up-and-Go	99	3	2
Functional Ambulation Categories	168.2	4	6.5
Berg Balance Scale	156.5	4.5	7
Five-Times Sit-to-Stand Test	266.6	9	7.88
Accelerometers, step activity monitors	264.8	9.5	7.15
Tinetti Falls Efficacy Scale	282.8	10	9.5
Rivermead Mobility Index	237.1	10.5	8.83
Functional Gait Assessment	272.2	11	6
Trunk Impairment Scale	273.7	11	8
Falls Efficacy Scale	303.8	11.1	6.75
Berg Balance Scale (3-Point Short Form)	280.3	12	8.5
Functional Reach Test	301.8	12.1	8.25
Balance Evaluation System Test	332.3	12.1	7
Rivermead Motor Assessment, Leg and Trunk Section	297.8	12.65	6.75
Mini Balance Evaluation System Test	310.7	13	8.75
Motor Assessment Scale Sitting Subscale	332.8	13.05	6
Four-Step Square Test	345.8	13.1	10
Fugl-Meyer Assessment Balance Subscale	322.8	13.75	6.5
Motor Assessment Scale for Lower Extremity	321.8	13.3	7.25
Step-Up Test	367.8	14	5.7
Postural Assessment Scale for Stroke Patients	389.8	15	5.38
**Activities (FAC<3)**			
Berg Balance Scale	45	2	1
Functional Ambulation Categories	56	2	3.25
Rivermead Mobility Index	76	4	2
Trunk Impairment Scale	78	4	3
Five-Times Sit-to-Stand Test	98	5	2
Motor Activity Scale	100	6	2
Postural Assessment Scale for Stroke	108	6	2.75
**Participation**			
Outcome measures on the participation level were rated within the ADL/ stroke-specific section			

Legend: ADL, Activities of Daily Living; FAC, Functional Ambulation Categories; IQR, Interquartile Range.

Table III: Rank list for Activities of Daily Living/ stroke-specific outcome measures

Outcome measure	Sum of ranks	Median rank	IQR
**Body functions**			
National Institutes of Health Stroke Scale	29	1	0
Visual Analogue Scale	58	2	0
**Activities**			
Functional Independence Measure	66.5	2	2
Barthel Index	71	2	2
Modified Rankin Scale	87.5	3	2
Modified Barthel Index	88.5	3	2
Lower Extremity Functional Scale	121.5	4.5	1
**Participation**			
Stroke Impact Scale	70	2	3
Stroke Impact Scale 16-Item Version	80.5	3	3.13
Stroke-Specific Quality of Life Scale	84.5	3	2
Caregiver Strain Index	101	3	2
European Quality of Life Scale	93	4	2

Legend: IQR, Interquartile Range.

2. Agreed measurements time points poststroke

Table IV: Agreement rates (%) for measurement time points for the upper and lower extremity sections by ICF-domain

Time poststroke / ICF-domain	Day 2±1	Day 7	Week 2	Week 4	Week 6	Week 12	Month 6	Every 6th month
**Body functions**	**82.6**	**78.3**	**78.3**	**91.3**	40.9	**78.3**	**91.3**	**73.9**
**Activities**	26.1	**91.3**	**65.2**	**87.0**	47.8	**82.6**	**73.9**	**69.6**
**Participation** *	6.3	30.4	52.2	30.4	26.1	**65.2**	43.5	**73.9**

Legend: Agreed measurement time points with a cut-off level of >70±5% are bold; *, Participation domain is the same across sections; ICF, International Classification of Functioning, Disability and Health.

Table V: Agreement rates (%) for measurement time points for the Activities of Daily Living/ stroke-specific section by ICF-domain

Time poststroke / ICF-domain	Day 2±1	Day 7	Week 2	Week 4	Week 6	Week 12	Month 6	Every 6th month
**Body functions**								
NIHSS	**69.7**	**75.0**	45.8	41.7	29.2	41.7	33.3	54.2
**Activities**								
BI	51.5	**76.0**	60.0	48.0	44.0	**76.0**	48.0	**72.0**
FIM	51.5	**69.2**	57.7	50.0	42.3	**76.9**	46.2	**69.2**
**Participation** *								
SIS	6.3	30.4	52.2	30.4	26.1	**65.2**	43.5	**73.9**

Legend: Agreement measurement time points with a cut-off level of >70±5% are bold; *, Participation domain is the same across sections; BI, Barthel Index; FIM, Functional Independence Measure; ICF, International Classification of Functioning, Disability and Health; NIHSS, National Institutes of Health Stroke Scale; SIS, Stroke Impact Scale.

3. Core set's outcome measures' details and clinimetric properties in stroke patients

Table VI: Details of the outcome measures for the upper extremity section

Outcome measure	Construct	Self-reported	Time (minutes)	Materials and costs	Validity (correlation coefficient, *r*)	Intra-rater/ Inter-rater reliability (ICC)	Responsiveness (MCID)
**Body functions**							
Fugl-Meyer Motor Assessment Upper Extremity Subscale[1,2]	Motor function	No	15	Reflex hammer, stopwatch, ball, cylinder, pen, paper-card ~30€	0.93 with ARAT[3,4]<br>0.92 with BBT[4]<br>0.86 with MI[4]<br>0.71 with GF[5]<br>0.58-0.68 with MAL[3]	0.95[2]/<br>0.66-1.0[6]	Acute to subacute:<br>9-10 points[2,7]<br>Chronic:<br>4.25 to 7.25 points[8]
**Activities**							
Action Research Arm Test[9,10]	Uni-manual dexterity	No	15	Test set ~600€	0.93 with FMMA-UE[3]<br>0.91 with SULCS[11]<br>0.81 with MI-UE[12]<br>0.79 with AMAT[13]	0.99[14]/<br>0.98[12,15]	Acute to subacute:<br>12 points dominant[16]<br>17 points non-dominant[16]<br>Chronic:<br>5.7 points[17]
**Participation**							
Stroke Impact Scale[18,19]	Health-related quality of life	Yes	5 *	Free	0.87 with BI[20]<br>0.81 with mRS[20]<br>0.48 with SF-36[20] *	No data/<br>0.94[20] *	Chronic:<br>9.2 points[21] (Strength Subscale)<br>5.9 points[21] (ADL Subscale)<br>17.8 points[21] (Hand Function Subscale)

Legend: *, only for Hand Function Subscale; ADL, Activities of Daily Living; AMAT, Arm Motor Ability Test; ARAT, Action Research Arm Test; BBT, Box and Blocks Test; BI, Barthel Index; FIM, Functional Independence Measure; FMMA-UE, Fugl-Meyer Motor Assessment Upper Extremity Subscale; GF, Grip Force; ICC, Intraclass Correlation Coefficient; MAL, Motor Activity Log; MAS, Motor Assessment Scale; MCID, Minimal Clinically Important Difference; MI-UE, Motricity Index Upper Extremity Subscale; mRS, modified Rankin Scale; NHPT, Nine-Hole Peg Test; SF-36, Short Form-36; SULCS, Stroke Upper Limb Capacity Scale.

Table VII: Details of the outcome measures for the lower extremity section

Outcome measure	Construct	Self-reported	Time (minutes)	Materials and costs	Validity (correlation coefficient, *r*)	Intra-rater/ Inter-rater reliability (ICC)	Responsiveness (MCID)
**Body functions**							
Fugl-Meyer Motor Assessment Lower Extremity Subscale[1,2]	Motor function	No	10	Reflex hammer, stopwatch ~20€	0.61 with GS[22]<br>0.77-0.89 with BI[23]<br>0.47 with FIM[6]	0.99[23]/<br>0.81-0.96[24]	Acute to subacute: No data<br>Chronic: 6 points[25]
10-Meter Walk Test[26]	Comfortable gait speed (applicable for FAC ≥3)	No	5	Stopwatch ~5€	0.76 with BI[27]<br>-0.68-(-0.83) with DGI[28]<br>-0.66-(-0.81) with FGA[28]<br>0.63 with BBS[29]<br>0.35 with FRT[30]	0.87-0.99[30]/<br>0.97-0.99[29–31]	Acute to subacute: 0.16 m/s[32]<br>Chronic: No data
**Activities**							
Berg Balance Scale[33]	Static/ dynamic balance in sitting and standing	No	20	Chair, stopwatch, measure tape, stepper ~30€	0.90-0.92 with FMMA-BS[34]<br>0.92-0.95 with PASS[34]<br>0.82-0.91 with MAS[34]	0.97[34]/<br>0.95-0.98[34,35]	Acute to subacute: 12.5 points[36]<br>Chronic: No data
Timed-up-and-Go[37]	Mobility/ balance/ gait (applicable for FAC ≥3)	No	5	Chair, stopwatch ~10€	-0.86-(-0,91) with GS[31]<br>0.86-0.90 with SCT[31]<br>0.92 with 6MWT[31]<br>-0.75 with CB&M[38]<br>-0.70 with BBS[31]	0.96[31]/<br>0.95[31]	Acute to subacute: No data<br>Chronic: No data
**Participation**							
Stroke Impact Scale[19,39]	Health-related quality of life	Yes	5 *	Free	0.53-0.83 with FIM[39]<br>0.82-0.84 with BI[39]<br>0.58 with SF-36[39] *	No data/<br>0.83-0.89[20]	Chronic:<br>5.9 points[21] (ADL Subscale)<br>4.5 points[21] (Mobility Subscale)

Legend: *, only for Mobility and ADL Subscales; 10MWT, 10-Meter Walk Test; 6MWT, 6-Minute Walk Test; ADL, Activities of Daily Living; BI, Barthel Index; BBS, Berg Balance Scale; CB&M, Community Balance and Mobility Scale; DGI, Dynamic Gait Index; FAC, Functional Ambulation Categories; FGA, Functional Gait Assessment; FIM, Functional Independence Measure; FMMA-BS, Fugl-Meyer Motor Assessment Balance Subscale; FRT, Functional Reach Test; GS, Gait Speed; ICC, Intraclass Correlation Coefficient; MCID, Minimal Clinically Important Difference; MAS, Motor Assessment Scale; m/s, meter per second; PASS, Postural Assessments Scale for Stroke; SCT, Stair Climbing Test; SF-36, Short Form-36.

Table VIII: Details of the outcome measures for the Activities of Daily Living/ stroke-specific section

Outcome measure	Construct	Self-reported	Time (minutes)	Materials and costs	Validity (correlation coefficient, *r*)	Intra-rater/ Inter-rater reliability (ICC)	Responsiveness (MCID)
**Body functions**							
National Institutes of Health Stroke Scale[40]	Neurological functions	No	10	Sheets with pictures and sentences<br>Free	0.48-0.62 with DWI-MRI[41]<br>0.60-0.85 with mRS[42,43]<br>-0.65 with BI[42,43]<br>-0.86 with GCS[43]	0.93[44]/ 0.92-0.96[44,45]	No data
**Activities**							
Barthel Index[46]	Basic ADL	Yes<br>No	5<br>20	Free	0.92-0.94 with FIM[15]<br>0.78-0.81 with FMMA[47]<br>0.89-0.91 with BBS[47]<br>0.59-0.66 with FAT[47]	No data/ 0.94[47]	Acute to subacute:<br>1.85 points (20-Point Version)[49]<br>Chronic: No data
Functional Independence Measure[48]	Basic ADL/ cognition	No	30-45	1590€ annually[§]	0.92-0.94 with BI[15]<br>0.77 with STREAM[49]<br>0.61-0.69 with POMA[50]	No data/<br>0.92-0.95[51]	Acute to subacute:<br>17 points (Motor Subscale)[52]<br>3 points (Cognitive Subscale)[52]<br>22 points (Total Scale)[52]<br>Chronic: No data
**Participation**							
Stroke Impact Scale[18,19]	Health-related quality of life	Yes	20 *	Free	0.87 with BI[20]<br>0.81 with mRS[20]<br>0.61 with SF-36[20]	No data/ 0.48-0.94[20]	Acute to subacute: No data<br>Chronic:<br>9.2 points (Strength Subscale)[21]<br>5.9 points (ADL Subscale)[21]<br>4.5 points (Mobility Subscale)[21]<br>17.8 points (Hand Function Subscale)[21]

Legend: *, time to administer complete scale; [§], according to information given by *Uniform Data System of Medical Rehabilitation*; ADL, Activities of Daily Living; BI, Barthel Index; BBS, Berg Balance Scale; DWI-MRI, Diffusion-Weighted Magnetic Resonance Imaging; FIM, Functional Independence Measure; FMMA, Fugl-Meyer Assessment; GCS, Glasgow Coma Scale; ICC, Intraclass Correlation Coefficient; MCID, Minimal Clinically Important Difference; mRS, modified Rankin Scale; POMA, Tinetti Performance Oriented Mobility Assessment; SF-36, Short Form-36; STREAM, Stroke Rehabilitation Assessment Measurement.

4. References

1. Fugl-Meyer AR, Jääskö L, Leyman I, Olsson S, Steglind S. The post-stroke hemiplegic patient. 1. A method for evaluation of physical performance. *Scand J Rehabil Med* (1975) **7**:13–31.

2. Sullivan KJ, Tilson JK, Cen SY, Rose DK, Hershberg J, Correa A, Gallichio J, McLeod M, Moore C, Wu SS, et al. Fugl-Meyer Assessment of sensorimotor function after stroke: standardized training procedure for clinical practice and clinical trials. *Stroke* (2011) **42**:427–432. doi:10.1161/strokeaha.110.592766

3. Wei XJ, Tong KY, Hu XL. The responsiveness and correlation between Fugl-Meyer Assessment, Motor Status Scale, and the Action Research Arm Test in chronic stroke with upper-extremity rehabilitation robotic training. *Int J Rehabil Res Int Z Rehabil Rev Int Rech Readaptation* (2011) **34**:349–356. doi:10.1097/MRR.0b013e32834d330a

4. Platz T, Pinkowski C, van Wijck F, Kim IH, di Bella P, Johnson G. Reliability and validity of arm function assessment with standardized guidelines for the Fugl-Meyer Test, Action Research Arm Test and Box and Block Test: A multicentre study. *Clin Rehabil* (2005) **19**:404–411. doi:10.1191/0269215505cr832oa

5. Kim H, Her J, Ko J, Park D, Woo J-H, You Y, Choi Y. Reliability, concurrent validity, and responsiveness of the Fugl-Meyer Assessment (FMA) for hemiplegic patients. *J Phys Ther Sci* (2012) **24**:893–899. doi:10.1589/jpts.24.893

6. Shelton FD, Volpe BT, Reding M. Motor impairment as a predictor of functional recovery and guide to rehabilitation treatment after stroke. *Neurorehabil Neural Repair* (2001) **15**:229–237. doi:10.1177/154596830101500311

7. Narayan Arya K, Verma R, Garg RK. Estimating the minimal clinically important difference of an upper extremity recovery measure in subacute stroke patients. *Top Stroke Rehabil* (2011) **18**:599–610. doi:10.1310/tsr18s01-599

8. Page SJ, Fulk GD, Boyne P. Clinically important differences for the upper-extremity Fugl-Meyer Scale in people with minimal to moderate impairment due to chronic stroke. *Phys Ther* (2012) **92**:791–798. doi:10.2522/ptj.20110009

9. Yozbatiran N, Der-Yeghiaian L, Cramer SC. A standardized approach to performing the Action Research Arm Test. *Neurorehabil Neural Repair* (2008) **22**:78–90. doi:10.1177/1545968307305353

10. Lyle RC. A performance test for assessment of upper limb function in physical rehabilitation treatment and research. *Int J Rehabil Res Int Z Rehabil Rev Int Rech Readaptation* (1981) **4**:483–492.

11. Houwink A, Roorda LD, Smits W, Molenaar IW, Geurts AC. Measuring upper limb capacity in patients after stroke: reliability and validity of the Stroke Upper Limb Capacity Scale. *Arch Phys Med Rehabil* (2011) **92**:1418–1422. doi:10.1016/j.apmr.2011.03.028

12. Hsieh CL, Hsueh IP, Chiang FM, Lin PH. Inter-rater reliability and validity of the Action Research Arm Test in stroke patients. *Age Ageing* (1998) **27**:107–113.

13. O'Dell MW, Kim G, Rivera L, Fieo R, Christos P, Polistena C, Fitzgerald K, Gorga D. A psychometric evaluation of the Arm Motor Ability Test. *J Rehabil Med* (2013) **45**:519–527. doi:10.2340/16501977-1138

14. van der Lee JH, Beckerman H, Lankhorst GJ, Bouter LM. The responsiveness of the Action Research Arm test and the Fugl-Meyer Assessment scale in chronic stroke patients. *J Rehabil Med* (2001) **33**:110–113.

15. Hsueh I-P, Lin J-H, Jeng J-S, Hsieh C-L. Comparison of the psychometric characteristics of the functional independence measure, 5-item Barthel Index, and 10-item Barthel Index in patients with stroke. *J Neurol Neurosurg Psychiatry* (2002) **73**:188–190.

16. Lang CE, Edwards DF, Birkenmeier RL, Dromerick AW. Estimating minimal clinically important differences of upper-extremity measures early after stroke. *Arch Phys Med Rehabil* (2008) **89**:1693–1700. doi:10.1016/j.apmr.2008.02.022

17. Simpson LA, Eng JJ. Functional recovery following stroke: Capturing changes in upper-extremity function. *Neurorehabil Neural Repair* (2013) **27**:240–250. doi:10.1177/1545968312461719

18. Duncan PW, Bode RK, Min Lai S, Perera S. Rasch analysis of a new stroke-specific outcome scale: The Stroke Impact Scale. *Arch Phys Med Rehabil* (2003) **84**:950–963. doi:10.1016/S0003-9993(03)00035-2

19. Duncan PW, Goldstein LB, Matchar D, Divine GW, Feussner J. Measurement of motor recovery after stroke. Outcome assessment and sample size requirements. *Stroke* (1992) **23**:1084–1089.

20. Carod-Artal FJ, Egido JA. Quality of life after stroke: The importance of a good recovery. *Cerebrovasc Dis* (2009) **27**:204–214. doi:10.1159/000200461

21. Lin K, Fu T, Wu C, Wang Y, Liu J, Hsieh C, Lin S. Minimal detectable change and clinically important difference of the Stroke Impact Scale in stroke patients. *Neurorehabil Neural Repair* (2010) **24**:486–492. doi:10.1177/1545968309356295

22. Nadeau S, Arsenault AB, Gravel D, Bourbonnais D. Analysis of the clinical factors determining natural and maximal gait speeds in adults with a stroke. *Am J Phys Med Rehabil* (1999) **78**:123–130.

23. Wood-Dauphinee SL, Williams JI, Shapiro SH. Examining outcome measures in a clinical study of stroke. *Stroke* (1990) **21**:731–739.

24. Sanford J, Moreland J, Swanson LR, Stratford PW, Gowland C. Reliability of the Fugl-Meyer Assessment for testing motor performance in patients following stroke. *Phys Ther* (1993) **73**:447–454. doi:10.1093/ptj/73.7.447

25. Pandian S, Arya KN, Kumar D. Minimal clinically important difference of the lower-extremity Fugl–Meyer Assessment in chronic stroke. *Top Stroke Rehabil* (2016) **23**:233–239. doi:10.1179/1945511915Y.0000000003

26. Graham JE, Ostir GV, Fisher SR, Ottenbacher KJ. Assessing walking speed in clinical research: A systematic review. *J Eval Clin Pract* (2008) **14**:552–562. doi:10.1111/j.1365-2753.2007.00917.x

27. Tyson S, Connell L. How to measure balance in clinical practice. A systematic review of the psychometrics and clinical utility of measures of balance activity for neurological conditions. *Clin Rehabil* (2009) **23**:824–840. doi:10.1177/0269215509335018

28. Lin J-H, Hsu M-J, Hsu H-W, Wu H-C, Hsieh C-L. Psychometric comparisons of 3 functional ambulation measures for patients with stroke. *Stroke* (2010) **41**:2021–2025. doi:10.1161/STROKEAHA.110.589739

29. Wolf SL, Catlin PA, Gage K, Gurucharri K, Robertson R, Stephen K. Establishing the reliability and validity of measurements of walking time using the Emory Functional Ambulation Profile. *Phys Ther* (1999) **79**:1122–1133.

30. Collen FM, Wade DT, Bradshaw CM. Mobility after stroke: Reliability of measures of impairment and disability. *Int Disabil Stud* (1990) **12**:6–9.

31. Flansbjer U-B, Holmbäck AM, Downham D, Patten C, Lexell J. Reliability of gait performance tests in men and women with hemiparesis after stroke. *J Rehabil Med* (2005) **37**:75–82. doi:10.1080/16501970410017215

32. Tilson JK, Sullivan KJ, Cen SY, Rose DK, Koradia CH, Azen SP, Duncan PW, for the Locomotor Experience Applied Post Stroke (LEAPS) Investigative Team. Meaningful gait speed improvement during the first 60 days poststroke: minimal clinically important difference. *Phys Ther* (2010) **90**:196–208. doi:10.2522/ptj.20090079

33. Berg KO, Wood-Dauphinee SL, Williams JI, Maki B. Measuring balance in the elderly: Validation of an instrument. *Can J Public Health Rev Can Sante Publique* (1992) **83** Suppl 2:S7-11.

34. Mao H-F, Hsueh I-P, Tang P-F, Sheu C-F, Hsieh C-L. Analysis and comparison of the psychometric properties of three balance measures for stroke patients. *Stroke* (2002) **33**:1022–1027.

35. Berg K, Wood-Dauphinee S, Williams JI. The Balance Scale: Reliability assessment with elderly residents and patients with an acute stroke. *Scand J Rehabil Med* (1995) **27**:27–36.

36. Song MJ, Lee JH, Shin WS. Minimal clinically important difference of the Berg Balance Scale scores in people with acute stroke. *Phy Ther Pehabil Sci* (2018) **7** (3), 102-108. doi:10.14474/ptrs.2018.7.3.102

37. Podsiadlo D, Richardson S. The Timed “Up & Go”: A test of basic functional mobility for frail elderly persons. *J Am Geriatr Soc* (1991) **39**:142–148. doi:10.1111/j.1532-5415.1991.tb01616.x

38. Knorr S, Brouwer B, Garland SJ. Validity of the Community Balance and Mobility Scale in community-dwelling persons after stroke. *Arch Phys Med Rehabil* (2010) **91**:890–896. doi:10.1016/j.apmr.2010.02.010

39. Duncan PW, Wallace D, Lai SM, Johnson D, Embretson S, Laster LJ. The Stroke Impact Scale version 2.0. Evaluation of reliability, validity, and sensitivity to change. *Stroke* (1999) **30**:2131–2140.

40. Brott T, Adams HP, Olinger CP, Marler JR, Barsan WG, Biller J, Spilker J, Holleran R, Eberle R, Hertzberg V. Measurements of acute cerebral infarction: A clinical examination scale. *Stroke* (1989) **20**:864–870. doi:10.1161/01.STR.20.7.864

41. Fink JN, Selim MH, Kumar S, Silver B, Linfante I, Caplan LR, Schlaug G. Is the association of National Institutes of Health Stroke Scale scores and acute magnetic resonance imaging stroke volume equal for patients with right- and left-hemisphere ischemic stroke? *Stroke* (2002) **33**:954–958.

42. Prasad K, Dash D, Kumar A. Validation of the Hindi version of National Institute of Health Stroke Scale. *Neurol India* (2012) **60**:40–44. doi:10.4103/0028-3886.93587

43. Schlegel D, Kolb SJ, Luciano JM, Tovar JM, Cucchiara BL, Liebeskind DS, Kasner SE. Utility of the NIH Stroke Scale as a predictor of hospital disposition. *Stroke* (2003) **34**:134–137.

44. Goldstein LB, Samsa GP. Reliability of the National Institutes of Health Stroke Scale: Extension to Non-Neurologists in the Context of a Clinical Trial. *Stroke* (1997) **28**:307–310. doi:10.1161/01.STR.28.2.307

45. Dewey HM, Donnan GA, Freeman EJ, Sharples CM, Macdonell RAL, McNeil JJ, Thrift AG. Interrater reliability of the National Institutes of Health Stroke Scale: Rating by neurologists and nurses in a community-based stroke incidence study. *Cerebrovasc Dis* (1999) **9**:323–327. doi:10.1159/000016006

46. Mahoney FI, Barthel DW. Functional evaluation: The Barthel Index. *Md State Med J* (1965) **14**:61–65.

47. Hsueh IP, Lee MM, Hsieh CL. Psychometric characteristics of the Barthel activities of daily living index in stroke patients. *J Formos Med Assoc Taiwan Yi Zhi* (2001) **100**:526–532.

48. Granger CV, Hamilton BB, Keith RA, Zielezny M, Sherwin FS. Advances in functional assessment for medical rehabilitation: *Top Geriatr Rehabil* (1986) **1**:59–74. doi:10.1097/00013614-198604000-00007

49. Ward I, Pivko S, Brooks G, Parkin K. Validity of the Stroke Rehabilitation Assessment of Movement Scale in acute rehabilitation: A comparison with the functional independence measure and Stroke Impact Scale-16. *PM R* (2011) **3**:1013–1021. doi:10.1016/j.pmrj.2011.08.537

50. Cavanagh SJ, Hogan K, Gordon V, Fairfax J. Stroke-specific FIM models in an urban population. *J Neurosci Nurs J Am Assoc Neurosci Nurses* (2000) **32**:17–21.

51. Ottenbacher KJ, Hsu Y, Granger CV, Fiedler RC. The reliability of the Functional Independence Measure: A quantitative review. *Arch Phys Med Rehabil* (1996) **77**:1226–1232. doi:10.1016/S0003-9993(96)90184-7

52. Beninato M, Gill-Body KM, Salles S, Stark PC, Black-Schaffer RM, Stein J. Determination of the minimal clinically important difference in the FIM instrument in patients with stroke. *Arch Phys Med Rehabil* (2006) **87**:32–39. doi:10.1016/j.apmr.2005.08.130
